# Decoding E-Cigarette Secrets: Unveiling Saliva and E-Liquid Composition through Fourier-Transform Infrared Spectroscopy

**DOI:** 10.1021/acsomega.4c08648

**Published:** 2025-03-14

**Authors:** Bruna Fernandes do Carmo Carvalho, Letícia Foiani, Gabriela Zucco, Natália de Carvalho Faria, Gabrielle Nepomuceno, Kethilyn Chris Sousa Silva, Roger Borges, Mônica Ghislaine Oliveira Alves, Mário Pérez-Sayáns, Herculano da Silva Martinho, Janete Dias Almeida

**Affiliations:** † Department of Biosciences and Oral Diagnosis, Institute of Science and Technology, São Paulo State University, São José dos Campos 12209, São Paulo, Brazil; ‡ Center of Natural and Human Sciences, Federal University of the ABC, Santo André 04829-310, São Paulo, Brazil; § Faculdade Israelita de Ciências da Saúde Albert Einstein, Hospital Israelita Albert Einstein, São Paulo 05653-000, São Paulo, Brazil; ∥ Oral Medicine, Oral Surgery and Implantology Unit, Faculty of Medicine and Dentistry, Universidade de Santiago de Compostela, Santiago de Compostela 15705 Spain; ⊥ Instituto de Investigación Sanitaria de Santiago, Santiago de Compostela 15706 Spain

## Abstract

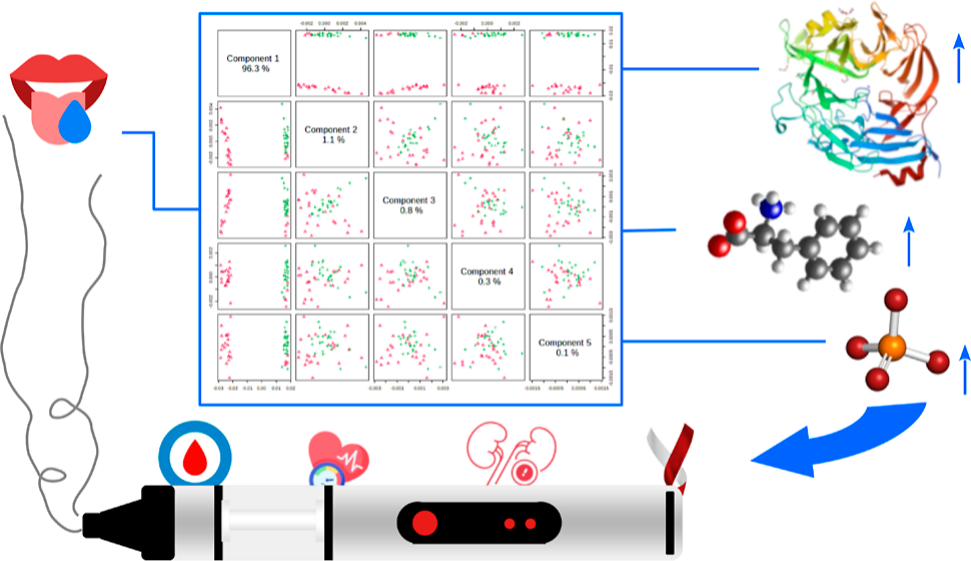

Electronic cigarettes (e-cigs), initially introduced as smoking cessation aids, have given rise to a new wave of nicotine dependence. A critical question that has emerged is the potential adverse effects of e-cig use on oral health, particularly how the vapor emitted from these devices may alter the salivary composition of users. Here, we investigate the salivary composition of e-cig users and analyze the e-liquids (flavorings) using Fourier-transform infrared (FTIR) spectroscopy. Saliva samples were categorized into two groups: e-cigarette users (25 individuals) and nonsmokers/nonusers (25 individuals). Additionally, 26 e-liquid samples used by the e-cig users were collected, with 17 obtained before use and 9 after use. The analysis provided reliable results in distinguishing between the two groups. Notably, partial least-squares discriminant analysis (PLS-DA) demonstrated a high degree of accuracy (>90%) in differentiating the sample groups. Our findings revealed a higher concentration of polysaccharides, aromatic amino acids, and inorganic phosphates, along with a lower concentration of esterases in the saliva of e-cigarette users. These alterations in salivary composition may be linked to an increased risk of type 2 diabetes, hypertension, cardiovascular diseases, kidney diseases, and tumor formation, having a negative impact on oral immunity. In contrast, no significant molecular or compositional changes were observed in the e-liquids after use. Our results underscore the importance of continued research into potential biomarkers and the long-term health effects associated with the growing prevalence of e-cigarette use as a form of nicotine consumption.

## 1 Introduction

Electronic cigarettes (e-cigs) were originally introduced as aids for smoking cessation but have since gained popularity as an alternative form of tobacco consumption. Their appeal is largely due to increased palatability and greater social acceptability, particularly among younger individuals. This shift to electronic nicotine delivery has fostered a new wave of nicotine dependence.^1^

The data collected systematically in Brazil over a 14 year period indicated a significant decrease in the prevalence of current cigarette smoking, heavy smoking, and passive smoking in the workplace among adults in Brazil from 2006 to 2019. While the reduction in the prevalence of smoking has decreased in intensity since 2015 (until 2019), the prevalence related to heavy smoking has intensified compared with the entire investigation period (2006–2019). Higher prevalence of cigarette smoking was systematically observed among men and those with a lower educational level. These same groups presented a smaller magnitude of reduction when compared, respectively, to women and individuals with higher educational levels, increasing their disadvantage.^2^

In spite of the lack of long-term population investigations, at first glance it is possible to argue that the vapor emitted from electronic devices can adversely affect oral health and alter the salivary profile of users, encompassing changes in physical–chemical composition, pH, total protein concentration, calcium, and phosphates.^3,4^

Saliva composition includes inorganic substances (such as sodium, potassium, calcium, magnesium, bicarbonate, and phosphates), DNA, RNA, immunoglobulins, proteins, enzymes, and nitrogenous products (like urea and ammonia).^5,6^ The composition of saliva can vary based on an individual’s physiological profile, encompassing factors like age, gender, diet, and habits such as sedentary lifestyle, alcohol consumption, and smoking.^7^ Investigation of its complex biofluid is promising for identifying biomarkers associated with both oral and systemic diseases. The smoking habits deserve particular attention since smoking proved to alter both saliva and crevicular fluid. Furthermore, it is well established that smoking is a pivotal risk factor contributing to oral dysbiosis and the emergence of dental injuries and mucosal lesions, notably oral cancer.^8,9^

Saliva plays a key role in flavor perception, which results from the combined effects of taste, aromatics, and chemical sensations in the oral cavity.^10^ By acting as a solvent, saliva enables food particles to dissolve and stimulates taste receptor cells within the taste buds of the lingual papillae (fungiform, foliate, and vallate).^11^ For e-cigarettes, aerosols generated from e-liquids—composed of propylene glycol (PG), vegetable glycerin (VG), nicotine, water, and flavorings^12^—interact with saliva upon inhalation. Although the e-liquid does not directly enter saliva, vapor-phase components dissolve through this interaction, redistributing the substances and potentially altering their concentrations, bioavailability, and flavor perception in the oral cavity.

Omics and vibrational techniques have emerged as robust complementary tools for diagnosing diseases or discerning changes in biological samples, including tissue, plasma, urine, and saliva.

Fourier transform infrared (FTIR) spectroscopy is an analytical technique used to identify and quantify chemical substances by measuring the absorption of infrared light at different wavelengths. In FTIR, a sample is exposed to infrared radiation, and the absorbed light is recorded to produce a spectrum that is unique to the molecular vibrations of the sample’s chemical bonds. The resulting spectrum can be used to identify the sample’s molecular composition. FTIR is widely used in medical diagnostics, particularly in the detection of various diseases and conditions. It can analyze biological samples (such as blood, urine, or tissues) for specific biomarkers associated with diseases, such as cancer, diabetes, or infections. FTIR is advantageous in diagnostics due to its nondestructive nature, rapid results, and ability to analyze complex biological samples with minimal sample preparation. It allows for the detection of subtle chemical changes in the sample that may indicate pathological conditions. Additionally, FTIR can be used in monitoring the progression of diseases or the effectiveness of treatments.^13,14^

Thus, Fourier-transform infrared (FTIR) spectroscopy enables fast and consistent assessment of structural changes in organic molecules.^15^ Recently, these spectral tools had been successfully applied to probe abuse drugs in biofluids, with blood serum (see, e.g., ref (16)) being a relevant ally in the opioid crisis.^17^

In this investigation, we aimed to identify the impact of e-cig usage on the molecular composition of saliva, considering potential physiologic risks and damage to the users’ health by using FTIR. In our prior research, our team utilized FTIR spectroscopy to successfully identify noteworthy differences in the overall composition of saliva between individuals who were actively smoking and those who had ceased smoking. Our investigation underscored rapid qualitative enhancements in the saliva of former smokers, particularly evident in collagen bands. These findings shed light on the favorable impact of smoking cessation on oral health within a relatively brief period.^18^ Our group has been working on the topic of healthcare providers and policymakers continuing to prioritize tobacco control efforts to reduce the burden of tobacco-related diseases and improve the well-being of our communities.^19^ In another preceding investigation, it was observed that users of e-cigs displayed genotoxicity and cytotoxicity markers in their oral mucosa cells. Notably, the identified damage cannot be solely attributed to e-cig use as a considerable proportion of participants also reported alcohol consumption and had a history of conventional cigarette use.^20^ Consequently, it becomes imperative to conduct further studies to comprehensively evaluate the long-term effects of e-cig usage. The expansion of our research endeavors will notably enhance our comprehension of how the use of an e-cig impacts the composition of saliva. This extension aims to draw meaningful parallels to the insights garnered from our investigations of traditional smoking. Such an exploration holds the potential to furnish valuable insights into the broader health ramifications of emerging smoking alternatives.

## 2 Materials and Methods

This investigation received approval from the Ethics Committee of the Institute of Science and Technology of São José dos Campos from São Paulo State University, São José dos Campos-SP, UNESP (ICT-UNESP) under protocol no. 4.397.780, CAAE 36911420.0.0000.0077.

### 2.1 Sample Selection

To recruit the participants, invitations were extended to other universities and through social media channels. Subsequently, 50 participants, exhibiting no visible clinical changes in the mucosa, were divided into two groups:

(a) Electronic cigarette group (EG): composed of 25 participants who consistently and exclusively use e-cigarettes for vaporization, with a minimum usage duration of at least 6 months,
(b) Control group (CG): composed of 25 individuals who are nonsmokers and non-e-cigarette users, matched in gender and age to the EG.

All participants underwent both extraoral and intraoral clinical examinations and responded to a questionnaire regarding their general health condition, smoking habits, and alcohol consumption.

#### 2.1.1 Inclusion Criteria

Participants aged 18 years and above willingly agreed to participate in the research by providing free and informed consent and adhered to the group-specific criteria.

#### 2.1.2 Noninclusion Criteria

Participants who engage in concurrent use of industrialized cigarettes and e-cigarettes (dual smokers), individuals undergoing treatment for autoimmune diseases, or those currently undergoing any form of surgical, radiotherapy, or chemotherapy oncological treatment were excluded.

#### 2.1.3 Exclusion Criteria

Cases lacking sufficient samples for laboratory analysis were excluded from the investigation. Participants were instructed to refrain from brushing their teeth or consuming any food for 2 h prior to the collection. Additionally, they were required to abstain from consuming alcohol for 12 h before the collection. To minimize oral debris, participants rinsed their mouths with distilled water for 1 min, 10 min before the collection.^3^ Doing so, we maximize the proportion of metabolic biomolecule content related to vaping with respect to food residues or other protein sources.

Unstimulated saliva was collected along 5 min in a quiet and isolated environment. Sialometry (mL/min) was determined by calculating the ratio of the volume of collected saliva in mL to the collection time in minutes.^21^ Seventeen e-liquid samples were collected before use for individuals in the EG group directly from the packaging (E-LB) and 9 after use (E-LA) from inside the electronic device. The collection was conducted by using sterile tips and microtubes. Subsequently, aliquots of all of the samples were prepared, hermetically sealed, and promptly stored in a −80 °C freezer.

### 2.2 Fourier-Transform Infrared (FTIR) Spectroscopy

After thawing at room temperature, aliquots of 1 μL of saliva were pipetted onto a platinum substrate (triplicate) and allowed to dry under controlled conditions. In order to preserve volatile (such as esters and aromatics) and low-fusion-point (such as esterases) residues in e-cig saliva, the drying process needed to be implemented in a very conservative way at a 20 °C temperature and at an estimated 80% relative humidity within a desiccator containing a NaCl-saturated solution. The spectra, spanning the range of 700 to 4000 cm^–1^, were acquired using the attenuated total reflectance (ATR) accessory with a ZnSe crystal on a Varian–Agilent 640-IR spectrometer, ensuring both precision and accuracy in the experimental setup. Samples of flavoring liquids of e-cigs were also analyzed.

### 2.3 Spectra Treatment and Analysis

The classical principal components analysis (PCA)^22^ was performed on mean-centered raw data to extract outliers and identify potential experimental bias. All spectral analysis steps were performed in R software using the ChemSpec vignette (R, 2018). The reduced Q-residual and T^2^ Hotelling’s statistics were used to identify outliers. Reduced Q-residuals measure the difference between a sample and its projection on the retained factors of the model. Examining reduced Q-residuals permits the detection of significant residual outliers. In contrast, Hotelling’s T^2^ value measures the variation in each sample within the model, indicating how far each sample is from the model’s center (scores = 0). It is a measure of score outliers. Raw spectral data were examined using the T^2^ Hotelling’s versus Q-residues (reduced) plot.

After outlier removal, all remaining spectra were preprocessed to make them statistically comparable. Baseline correction was performed using a curve-fitting method proposed by Lieber and Mahadevan-Jansen,^23^ based on a least-squares polynomial curve fitting. All spectra were normalized and scaled using probabilistic quotient normalization.^24^ The partial least-squares discriminant analysis (PLS-DA) was then carried out. It is a multivariate supervised approach that predicts class membership using the linear regression of original data. We employed the plsr function from the R pls package^25^ to perform the PLS regression. The classification and cross-validation were performed using the caret package equivalent wrapper function.^26^ A permutation test was used to evaluate the performance of class discrimination. A PLS-DA model was constructed between the data and the permuted class labels in each permutation using the optimal number of components determined via leave-one-out cross-validation for the model based on the original class assignment. The classification accuracy, *R*^2^, and *Q*^2^ were used to assess the performance of class discrimination.^13^ In the PLS-DA model, two quantifiers were utilized to assess the relevance of the vibrational band frequency. The first, variance importance projection (VIP) scores, is a weighted sum of squares of the PLS loadings that considers the amount of explained spectral intensity fluctuation in each dimension. The other measure of relevance is based on the weighted sum of the PLS regression. The weights are determined by dividing the sums of squares by the number of PLS components. The exact number of predictors will be constructed for each group in a multiple-group analysis, and the average of the feature coefficients will be utilized to represent the overall coefficient-based relevance. The receiver operating characteristic (ROC) analysis was utilized to assess discriminating performance, with the area under the ROC curve (AUC) being used as the summary index. AUC > 0.80 is often obtained in tests with good discriminating power.^27^

### 2.4 Contact Angle

The influence of e-cigarette and e-liquid use on the physical–chemical properties of saliva was evaluated by measuring the contact angle of the saliva, thereby understanding how such use behavior could impact the hydrophilicity of saliva. The contact angle was measured in a Phoenix 300 manual contact angle analyzer (SEO, Surface Electro Optics). One μL of the sample was pipetted on a platinum substrate, the imaging of the droplet was captured by the camera, and the contact angle was automatically measured by the equipment considering the average angle between the left and right angles of the droplet.

## 3 Results

**Figure 1:**
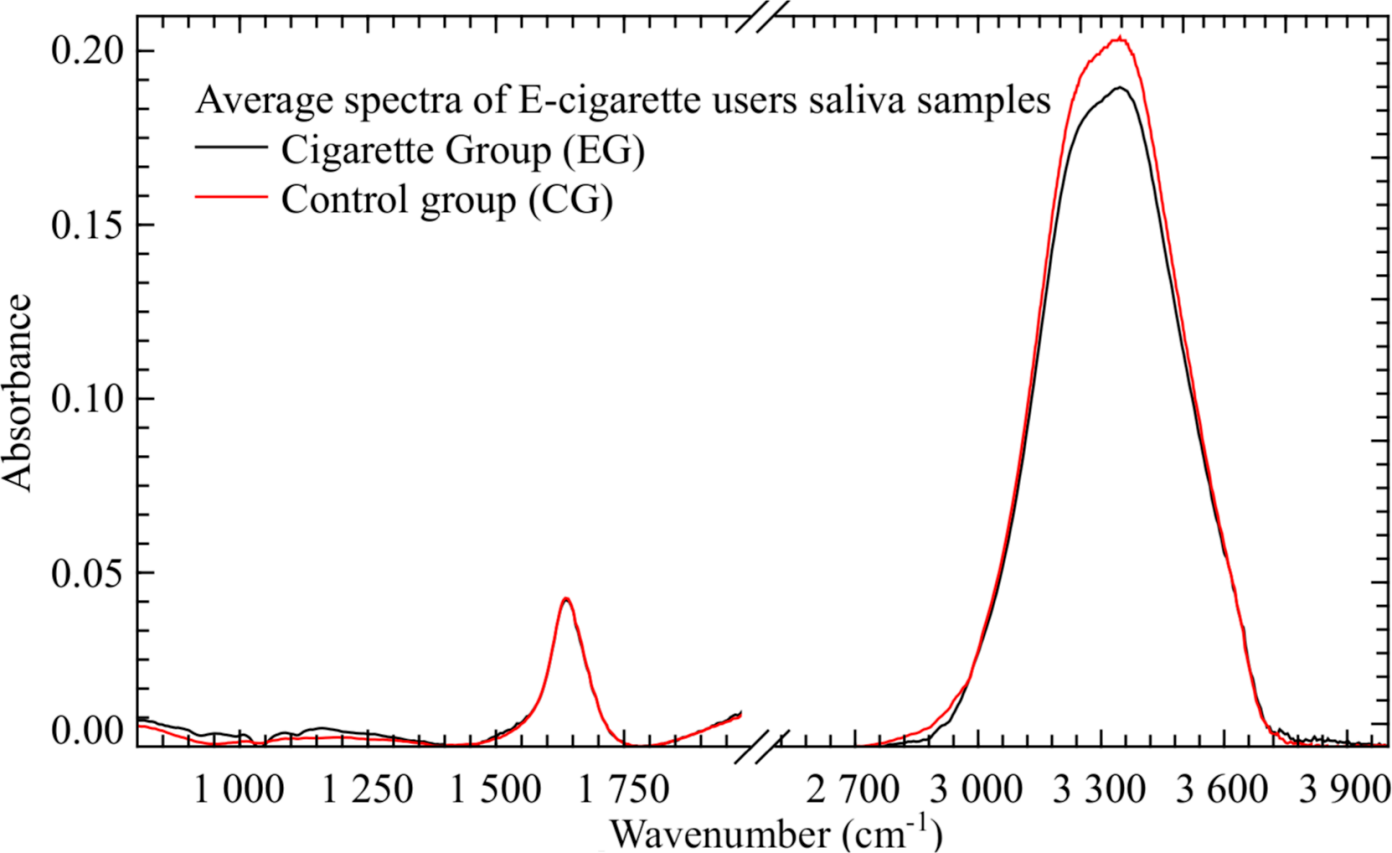
Average spectra of EG and CG groups of samples.

The average spectrum of the saliva samples in the EG and CG groups is shown in Figure 1. Raw spectra are presented in Figure S1 (Supporting Information). Bands at 1600 and 3300 cm^–1^ dominate the spectra. Those bands are related to amide I vibrations related to peptidic bonds in proteins and NH/OH stretching related to saccharides, while the last clearly decreased its intensity in the EG group. When comparing our spectra to those published previously in the literature, it is possible to conclude that all reported bands are present. However, there is an important difference in our case related to the relatively lower intensity of protein bands, which changed the general aspects of saliva FTIR spectra when compared to other works. This fact is related to a key aspect of our methodology: to minimize oral debris, all participants rinsed their mouths with distilled water for 1 min just 10 min prior to saliva collection. In this way, the available amount of proteins from free food residues or other sources was diluted in order to observe in experiments only changes related to metabolic processes. We also notice that the spectral region 800–1500 cm^–1^ appeared more intense in the EG group. In the following, we will separate the spectral analyses in the fingerprint (800–2000 cm^–1^) and high-wavenumber (2500–4000 cm^–1^) spectral regions.

**Figure 2:**
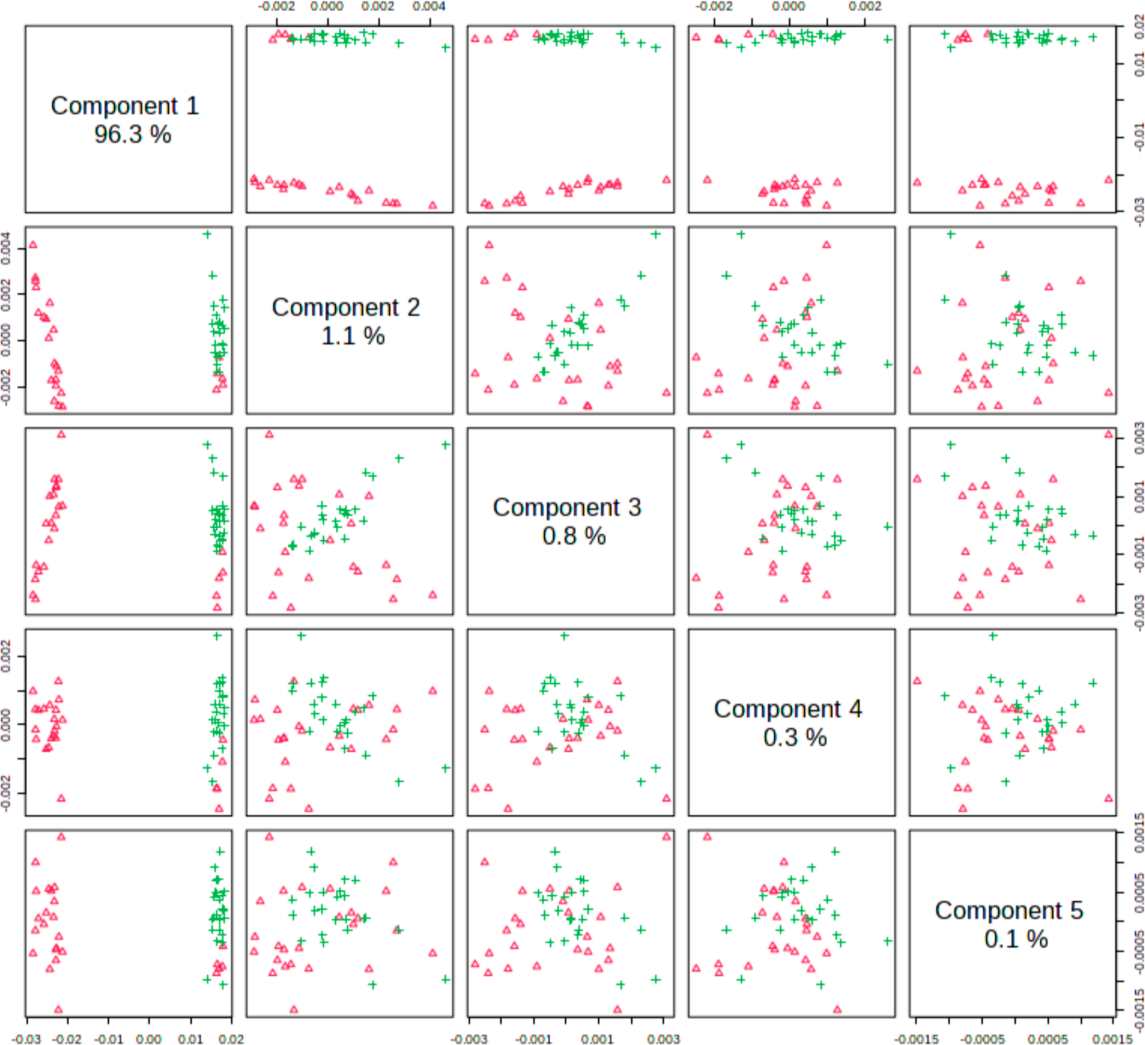
Pairwise scores of control group (CG, red triangles) and cigarette group (EC, green cross) individuals in the fingerprint region (800–2000 cm^–1^). The number in the diagonal axis shows the explained variance among the evaluated groups by each component.

**Figure 3:**
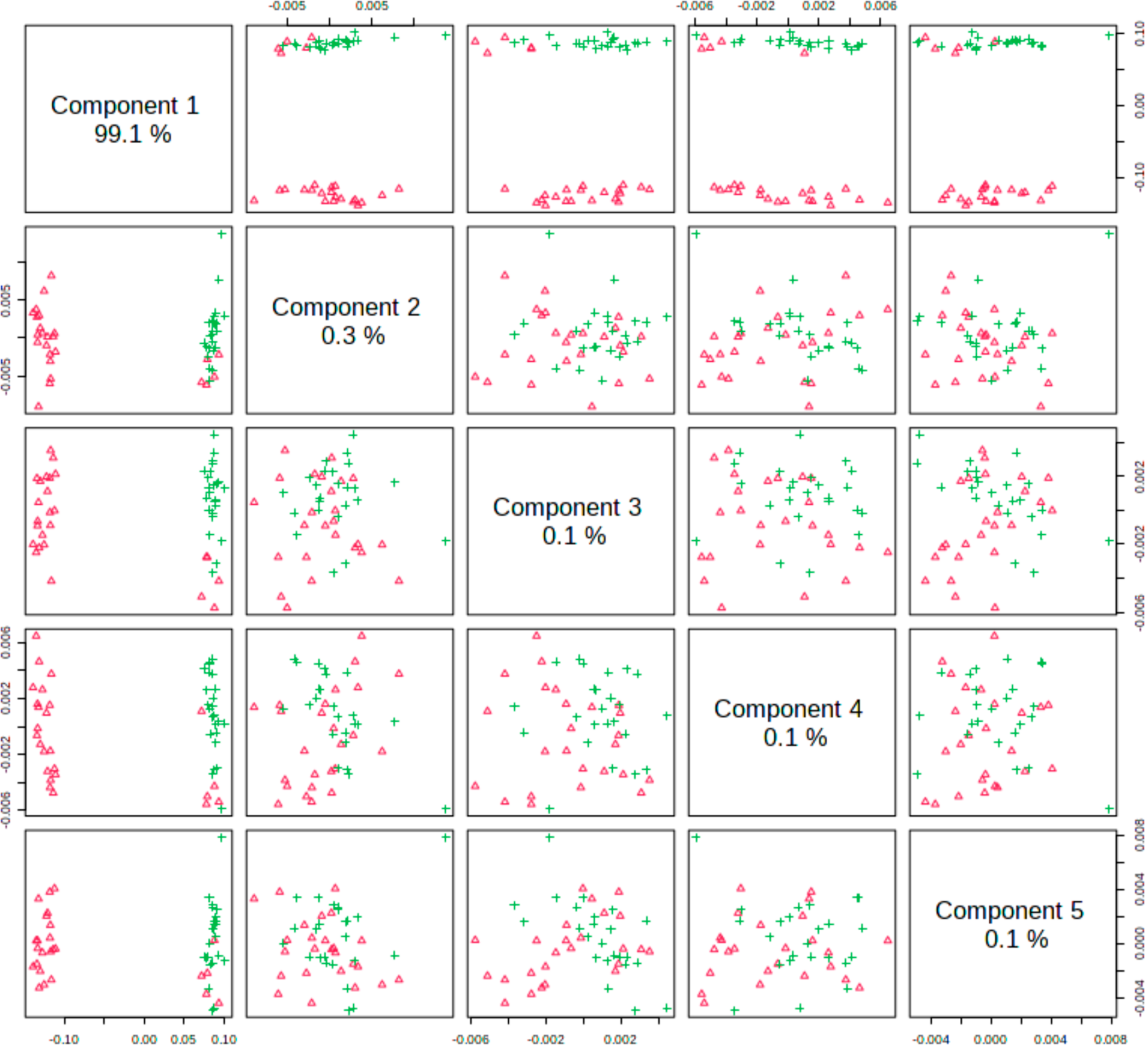
Pairwise scores of control group (CG, red triangles) and cigarette group (EC, green cross) individuals in the high-wavenumber region (2500–4000 cm^–1^). The number in the diagonal axis shows the explained variance among the evaluated groups by each component.

When the sample spectra were analyzed by PLS-DA, they presented very good discrimination between CG and EG groups in the fingerprint and in the high-wavenumber regions. Figure 2 shows the pairwise score plots in the fingerprint region. It is clear the discrimination among CG and EG for combinations including component 1. A similar trend was observed in the high-wavenumber region (Figure 3).

**Figure 4:**
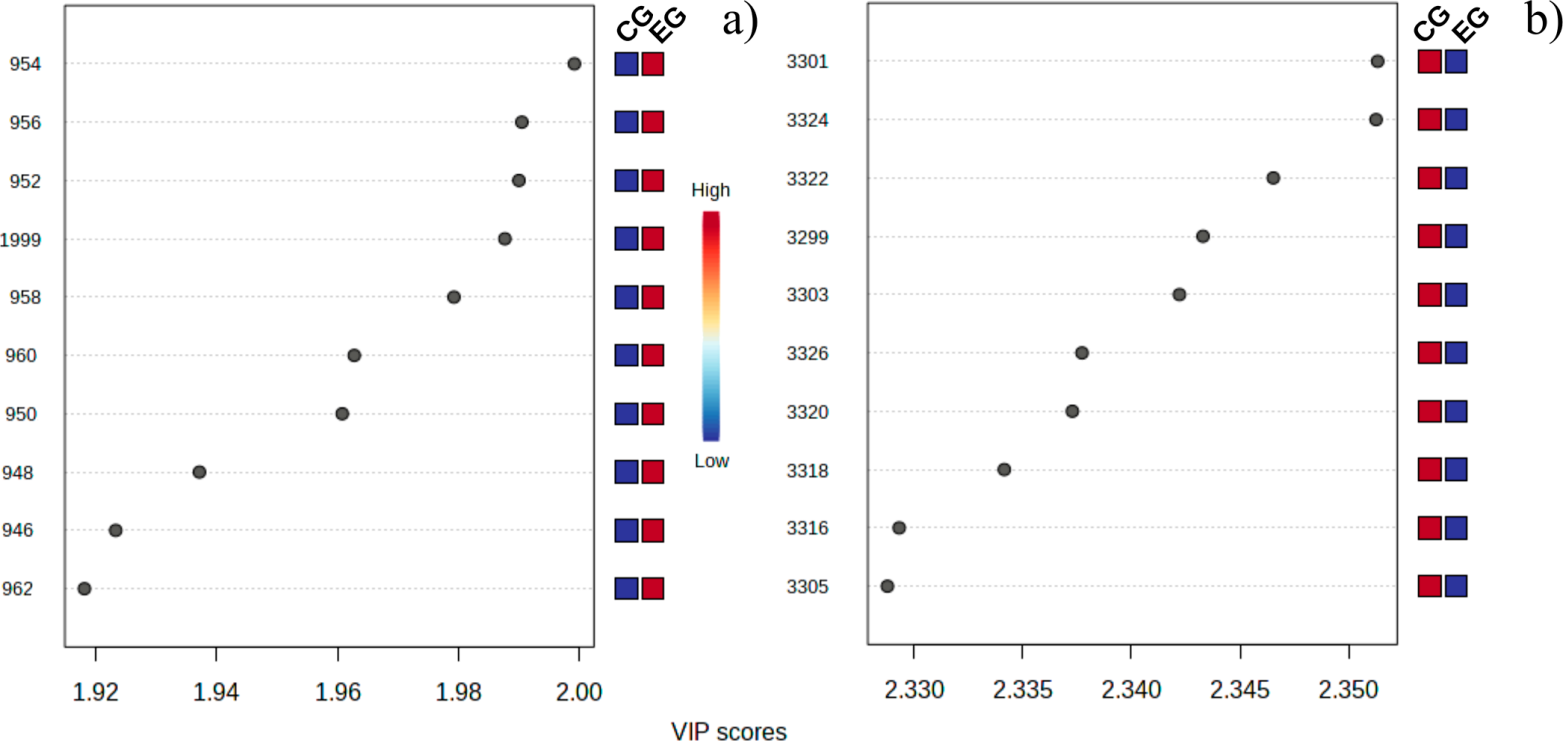
VIP scores of the control group (CG) and electronic cigarette group (EC) for fingerprint (a) and high-wavenumber (b) regions. The red color represents high intensity, while the blue represents low intensity.

Figure 4 shows the PLS-DA VIP Scores for the most discriminating bands in the fingerprint (Figure 4a) and high-wavenumber (Figure 4b) regions. Notably, the fingerprint spectral region exhibited larger intensities in the EG group as opposed to the CG, which is a discernible difference when just comparing spectral averages (Figure 1). Table 1 presents the assignments of the bands listed on VIP for both EG and GC, enabling further comparative analysis and molecular interpretation of the data. We notice that the accuracy of discrimination in the fingerprint and high-wavenumber regions was greater than 89% in both cases. *R*^2^ and *Q*^2^ parameters were also at good levels (greater than 70%).

**Table 1:** Assignments for the Main Vibrational Bands^9,28,29^ Found in the Electronic Cigarette Group (EC)/Control Group (CG) of Saliva and E-Liquid before (E-LB) and after (E-LA) Use, Considering an Interval of ±5 cm^–1^

ν (cm^–1^)	CG	EC	assignment, biomolecule
945	lower	higher	C–O stretching, polysaccharides
955	lower	higher	C–H (aromatic bonds) out-of-plane bending, aromatic amino acids residues as from phenylalanine
960	lower	higher	phosphate ion (PO_4_^–3^) symmetric stretching, inorganic phosphates
1155	lower	higher	(PO_2_^–2^) asymmetric stretching, inorganic phosphates
2000	lower	higher	(PO_2_^–2^) asymmetric stretching, inorganic phosphates
3300–3366	higher	lower	O–H stretching, esters as in esterases

ν (cm^–1^)	E-LB	E-LA	assignment, biomolecule
3235	lower	higher	O–H stretching, hydrogen bonding network
3252–3260	lower	higher	O–H stretching, esters

**Figure 5:**
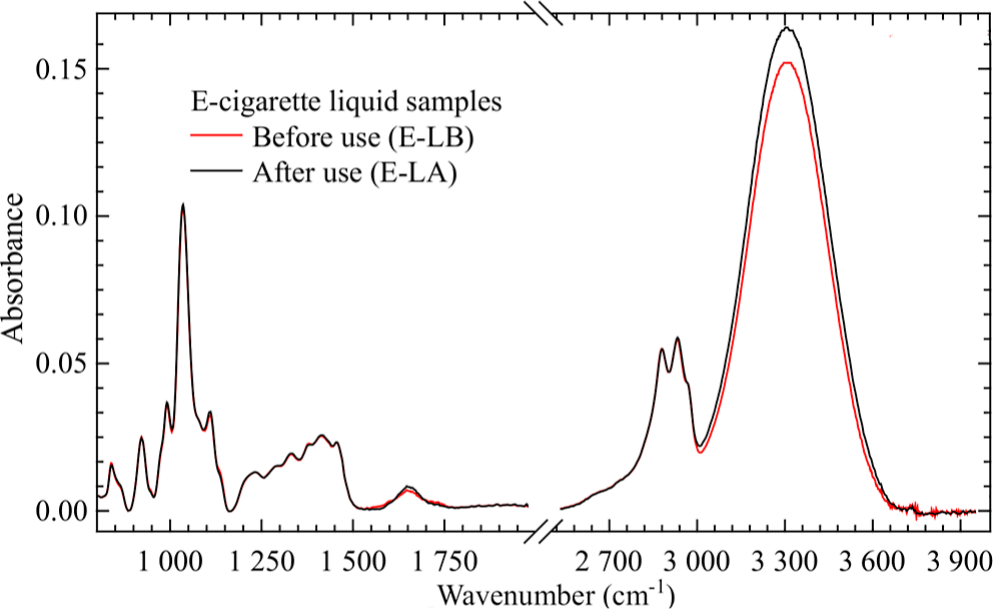
Average spectra of E-LA and E-LB groups of samples.

The average spectra of the vape liquids before (E-LB) and after (E-LA) use are shown in Figure 5. The most distinctive difference occurred between 3000 and 3600 cm^–1^, where the E-LB intensity decreased.

**Figure 6:**
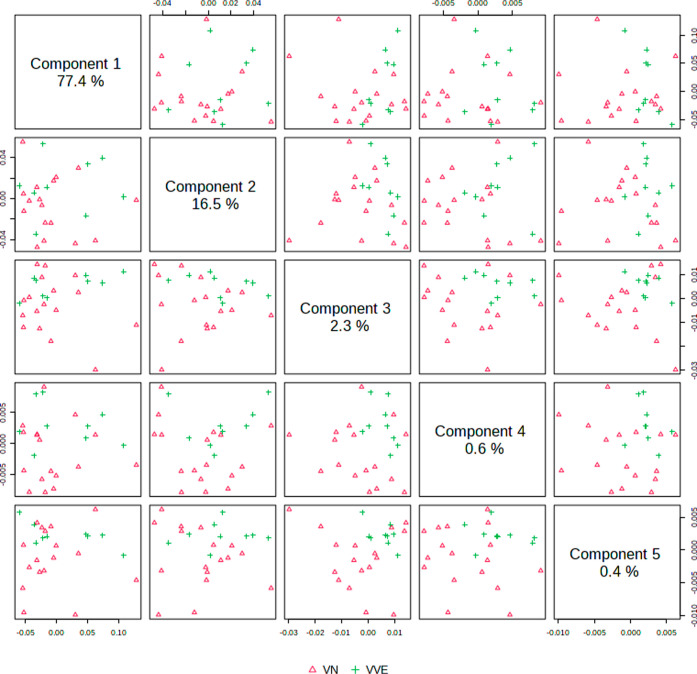
Pairwise scores of PLS-DA of e-liquid samples before (E-LB, red triangles) and after use (E-LA, green crosses) in the fingerprint spectral region. The number in the diagonal axis shows the explained variance among the evaluated groups by each component.

After analyzing vape liquid spectral data by PLS-DA, we noticed a poor discrimination using fingerprint regions. The pairwise score plot in this case (Figure 6) did not present distinctive grouping, which manifested in poor performance indicators (accuracy ⟨60%, *R*^2^ < 30%, *Q*^2^ < 0).

**Figure 7:**
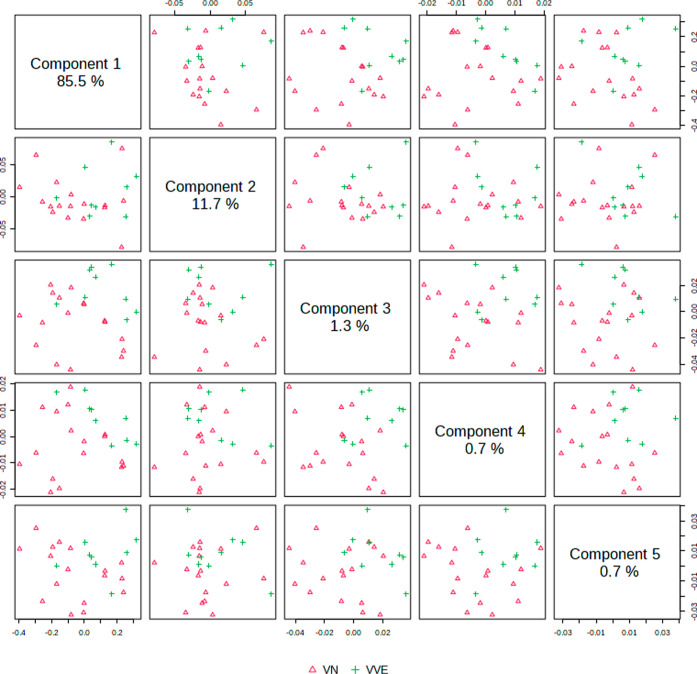
Pairwise scores of PLS-DA of e-liquid samples before (E-LB, red triangles) and after use (E-LA, green crosses) in the high-wavenumber spectral region. The number in the diagonal axis shows the explained variance among the evaluated groups by each component.

On the other hand, discrimination was clearly established for high-wavenumber. The pairwise score plots in Figure 7 show separation of E-LB and E-LA groups for pairs with components 1, 2, and 3. The indicators of group discrimination performance in this case were greater than 90%.

**Figure 8:**
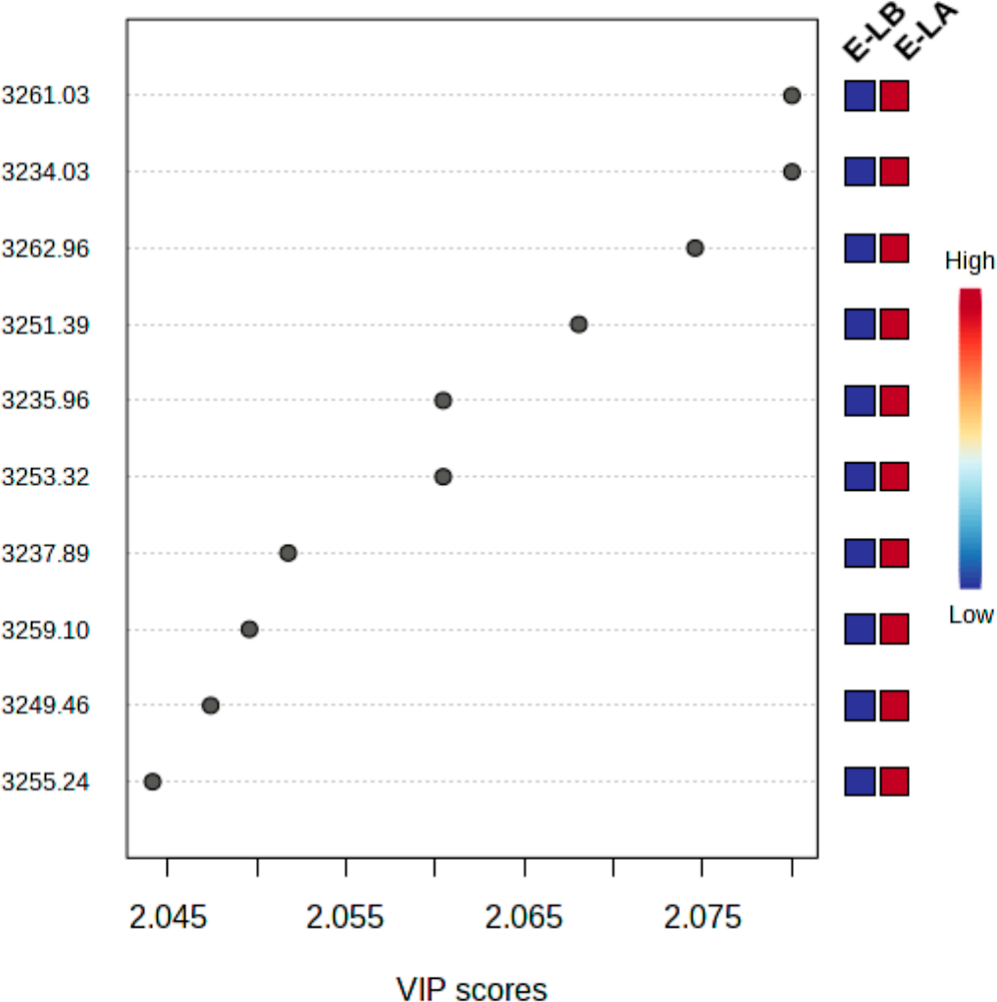
VIP scores of e-liquid before use (E-LB) and e-liquid after use (E-LA) in the high-wavenumber regions. The red color represents high intensity, while the blue represents low intensity.

The VIP scores for the most discriminating bands in the high-wavenumber for vape liquid discrimination are shown in Figure 8. The corresponding band assignments are summarized in Table 1.

Our analyses demonstrated the effective discrimination of saliva sample groups where a minimum set of significant bands played a crucial role. The developed statistical models exhibited excellent discriminatory performance with accuracy and *R*^2^, enabling their use for predicting the classification of new samples. Similarly, for vape liquid samples, only the high-wavenumber region achieved remarkable accuracy and *R*^2^ surpassing 90%.

**Figure 9:**
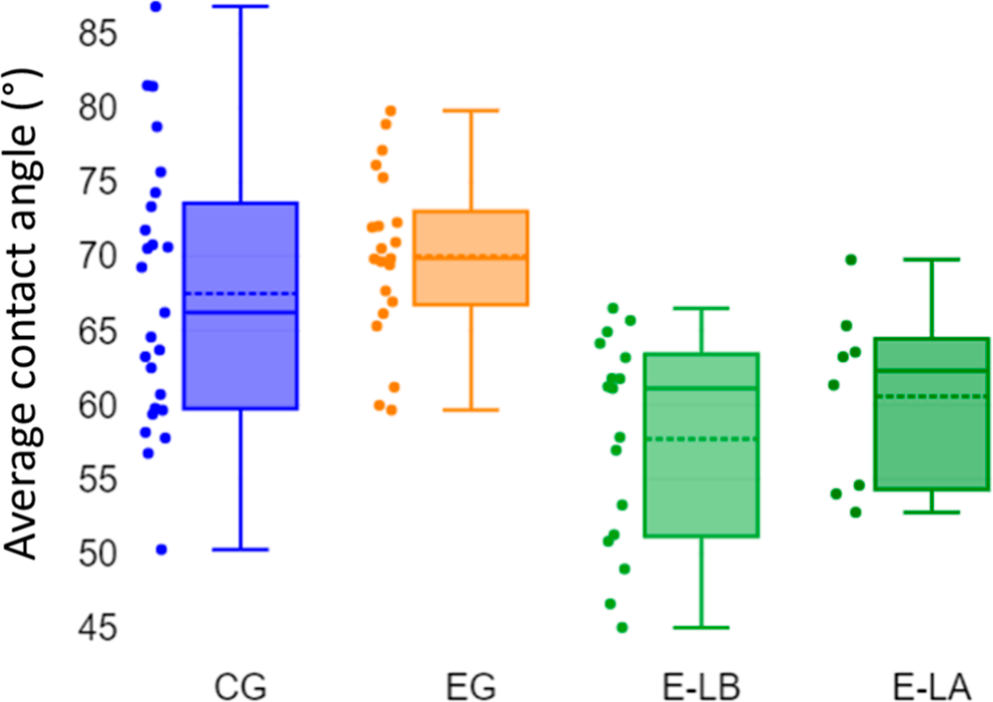
Average contact angles of the different experimental groups. The * symbol denotes the significant difference (*p* < 0.05) of the E-LB groups in regard to the CG and EG groups.

Figure 9 shows the result of contact angle measurements of the CG, EG, E-LA, and E-LB. First, it was noticed that the E-LA and E-LB groups did not show a significant difference besides showing similar mean contact angles (60.6 ± 6.1 and 57.7 ± 7.0, respectively). Second, a similar trend was noticed for CG and EG, whose mean contact angles were 67.5 ± 9.0 and 70.1 ± 8.1, respectively. However, the EG group showed less dispersion in the collected data than the CG group. When saliva samples (EG and CG) were compared to E-LB, a significant difference was noticed (*p* – value < 0.05), where those samples from e-liquid were less hydrophilic than those from saliva.

## 4 Discussion

Our FTIR results indicated that salivary composition changed for e-cig users. Observing the summary of saliva data in Table 1, we observed an increased content of polysaccharides, aromatic amino acid residues such as phenylalanine, and inorganic phosphates in e-cig users’ saliva. On the other hand, bands associated with esters decreased on e-cig saliva. We argue that an abundance of polysaccharides may be linked to enzymatic dysfunction resulting from the consumption of e-cigarettes. This dysfunction could potentially have a direct impact on the health. It has been demonstrated that e-cigarette aerosol exposure can alter the composition and function of saliva, including antioxidant capacity and enzymatic activity (e.g., α-amylase), which plays a crucial role in carbohydrate metabolism.^30,31^ Additionally, changes in microbial communities induced by e-cigarette use may contribute to alterations in polysaccharide levels due to their involvement in carbohydrate metabolism and biofilm formation.^32^ These disruptions could indirectly support the hypothesis of an association among e-cigarette use, enzymatic dysfunction, and polysaccharide abundance. This fact is of special concern since t is well-established that a diet characterized by high consumption of processed foods, added sugars, and refined carbohydrates significantly contributes to the increasing prevalence of type 2 diabetes, hypertension, and cardiovascular diseases.^33^

Aerosols generated from e-liquids, which are composed of propylene glycol, vegetable glycerin, nicotine, water, and flavorings,^12^ interact with saliva upon inhalation. Although the e-liquid does not directly enter saliva, vapor-phase components dissolve through this interaction, redistributing the substances and potentially altering their concentrations, bioavailability, and flavor perception in the oral cavity.

In fact, e-cig use has been associated with several changes in the composition of saliva, which reflect the potential impact of vaping on oral health and overall physiology. Research on this topic is still evolving, but some notable reported changes in the saliva composition of e-cigarette smokers include the following:

### 4.1 Increased Biomarkers of Oxidative Stress

E-cigarette use can lead to an increase in the number of oxidative stress markers in saliva. The inhalation of e-cigarette vapor introduces chemicals that can react with saliva, leading to the formation of free radicals and the depletion of antioxidants in the mouth. This may increase the risk of tissue damage and inflammation in the oral cavity.^34^

### 4.2 Changes in pH Levels

Saliva pH has been observed to shift slightly in e-cigarette users, often becoming more acidic. This acidity can contribute to enamel erosion and may influence the growth of the oral bacteria. Changes in pH could be related to the specific ingredients used in e-liquids, such as nicotine, flavoring agents, and acids.^35^

### 4.3 Altered Salivary Proteins

Research indicates that the composition of proteins in saliva may change in e-cigarette users, particularly in terms of proteins related to inflammation and immune responses. For example, there may be increases in certain cytokines and enzymes that are markers of inflammatory or immune reactions, suggesting that e-cigarette use could trigger local inflammation in the oral tissues.^35^

### 4.4 Increased Levels of Nicotine

As expected, e-cigarette users have higher levels of nicotine in their saliva compared to nonsmokers. The concentration of nicotine in saliva depends on factors such as the type of e-cigarette device used, the nicotine content in e-liquids, and the frequency of use. This can affect the oral cavity and the surrounding tissues, potentially contributing to a higher risk of gum disease.^36^

### 4.5 Presence of Harmful Chemicals

E-cigarette vapor contains a variety of chemical compounds, many of which can be detected in saliva. These include propylene glycol, glycerin, flavoring agents, and aldehydes. Some studies report that these compounds may linger in the mouth, contributing to changes in oral microbiota and potentially leading to oral irritation, dry mouth, and an increased risk of infections.^35^

### 4.6 Altered Oral Microbiota

Research has also shown that e-cigarette use may alter the balance of bacteria in the oral microbiome. Vaping has been linked to a reduction in beneficial bacteria and an increase in harmful bacterial species, potentially leading to oral diseases such as gingivitis or periodontitis.^37^

### 4.7 Dry Mouth and Reduced Salivary Flow

E-cigarette smokers often report experiencing dry mouth (xerostomia) and a reduction in salivary flow, which may be caused by nicotine’s vasoconstrictive effects. Reduced salivation can lead to a higher risk of cavities, gum disease, and oral discomfort beyond increasing the concentration of toxic compounds.^37^

We can mention that the observed increased aromatic amino acid content is probably related to increased bacterial metabolism, where phenylacetate is an important product, as observed in studies involving periodontitis.^38^ Additionally,^39^ it revealed a robust correlation between salivary phenylacetate levels and various oral health variables, including a noteworthy association with a 5 year risk of tooth loss, highlighting its potential as an indicator of oral health and a predictor of long-term dental outcomes. Moreover, the elevated levels of inorganic phosphate found in e-cigarette saliva could further increase risks of cardiovascular and kidney diseases and tumor formation, with potential associations to obesity.^40^ Conversely, the decrease in ester content raises significant concern as esterases—produced by mononuclear phagocytic cells—are vital for maintaining oral health and immune defense in both healthy and inflamed gingiva.^41^ This reduction may reflect a compromised immune response due to reduced phagocytic cell availability among e-cigarette users. Given the oral environment’s exposure to antigens from both food and the microbiota, diminished esterases become particularly concerning in the context of altered oral immunity, highlighting the need to investigate how dietary habits and e-cigarette use together shape oral and systemic health outcomes. On the other hand, FTIR spectra for the E-LB and E-LA groups did not show significant differences in the fingerprint region while showing relevant differences in the high-wavenumber region. The used e-cig liquid presented an increased hydrogen bonding network and ester content probably related to the burning process.

The difference in the data dispersion between the CG and EG groups observed in contact angle data deserves some attention. First, the dispersion in contact angle values of CG individuals may be attributed to variations in protein concentration, lipolysis, proteolysis, amylolysis, lipocalin concentration, lysozyme activity, total antioxidant status, and uric acid concentrations, which can either change the hydrophilic character of saliva by themselves or can yield chemical reactions whose products can lead to changes in hydrophilicity.^42,43^ Second, some works from the literature have already shown that continuous exposure of the oral cavity to e-cigarettes can harm the cells and mucosa of oral tissues.^44^ Taken together, the results from Figure 7 emphasize that the exposure of the oral cavity to e-cigarettes may restrict the composition of saliva, affecting its hydrophilic character. In fact, the results displayed in Table 1 showed that exposure of the oral cavity to e-cigarettes led to a decrease in amide-containing molecules such as proteins and enzymes. Therefore, it is reasonable to assume that enzymatic malfunction or decrease in protein concentration led to less variability among the hydrophilic character of the saliva of individuals.

## 5 Conclusions

Our FTIR results indicated that salivary composition changed due to e-cig usage. We observed an increased content of polysaccharides, aromatic amino acid residues such as phenylalanine, and inorganic phosphates in e-cig users’ saliva. On the other hand, bands associated with esters decreased on e-cig saliva. Unfortunately, these changes are correlated to enzymatic dysfunction, impacting the carbohydrate metabolism, resulting in an abundance of its biocomponent. This could increase the prevalence of type 2 diabetes, hypertension, and cardiovascular diseases. Higher incidence of periodontitis, jointly with potential associations to obesity, are also aspects of concern. Moreover, the observed depleted esterase availability had a direct negative impact on oral immunity. On the other hand, our findings suggest a relative stability in the molecular and compositional profile of e-cigarette liquids postuse.

These insights contribute significantly to our understanding of the physiological consequences of e-cig use and underscore the importance of ongoing investigations into potential biomarkers and long-term health effects associated with this increasingly prevalent form of nicotine consumption.

## Supporting Information Available

Raw FTIR data acquired in this investigation (PDF)
