# Supplementary material for: Decoding E-Cigarette Secrets: Unveiling Saliva and E-Liquid Composition through Fourier-Transform Infrared Spectroscopy
Source: ACS Omega. 2025 Mar 14;10(12):11911–21. doi: 10.1021/acsomega.4c08648 (PMC11966275; doi:10.1021/acsomega.4c08648)
Supplement: Supplementary file 1 — ao4c08648_si_001.pdf [file ao4c08648_si_001.pdf]

Decoding e-cigarette secrets: unveiling saliva and e-liquid composition through Fourier-transform infrared spectroscopy

Bruna Fernandes do Carmo Carvalho,[†] Letícia Foiani,[‡] Gabriela Zucco,[†] Natália de Carvalho Faria,[†] Gabrielle Nepomuceno,[‡] Kethilyn Chris Sousa Silva,[†] Roger Borges,[‡¶] Mônica Ghislaine Oliveira Alves,[†] Mário Pérez-Sayáns,[§,‖] Herculano da Silva Martinho,[*,‡] Janete Dias Almeida[*,†]

[†]*Department of Biosciences and Oral Diagnosis, Institute of Science and Technology, São Paulo State University, São José dos Campos-SP, Brazil*

[‡]*Center of Natural and Human Sciences, Federal University of the ABC, Santo André-SP, Brazil*

[¶] *Faculdade Israelita de Ciências da Saúde Albert Einstein, Hospital Israelita Albert Einstein. São Paulo-SP, Brazil*

[§]*Oral Medicine, Oral Surgery and Implantology Unit, Faculty of Medicine and Dentistry, Universidade de Santiago de Compostela. Santiago de Compostela, Spain*

[‖]*Instituto de Investigación Sanitaria de Santiago, Santiago de Compostela, Spain*

E-mail: herculano.martinho@ufabc.edu.br; janete.almeida@unesp.br

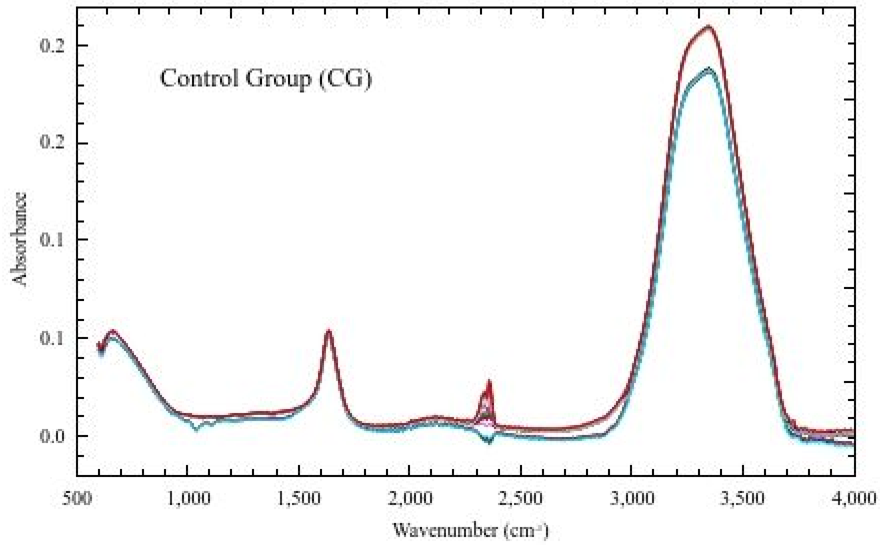

S1. Raw FTIR for saliva samples in control group.

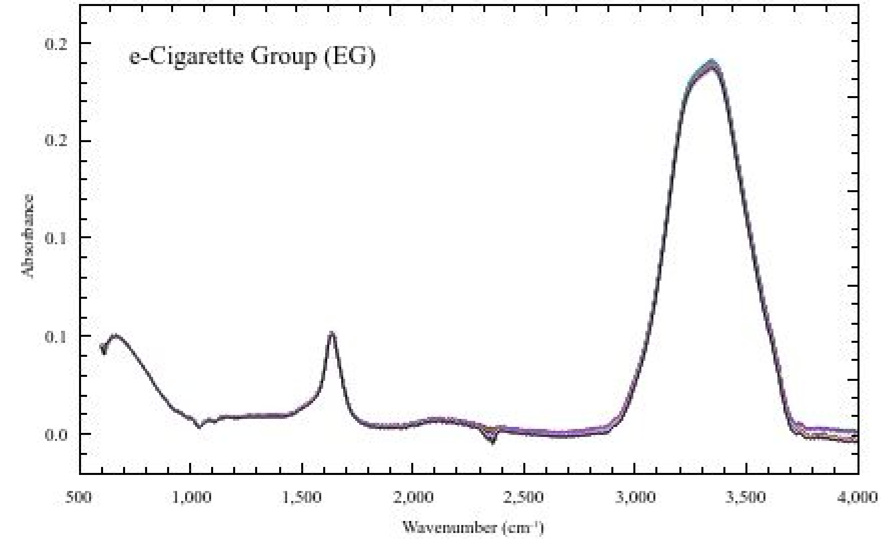

S2. Raw FTIR for saliva samples in the e-cig group.

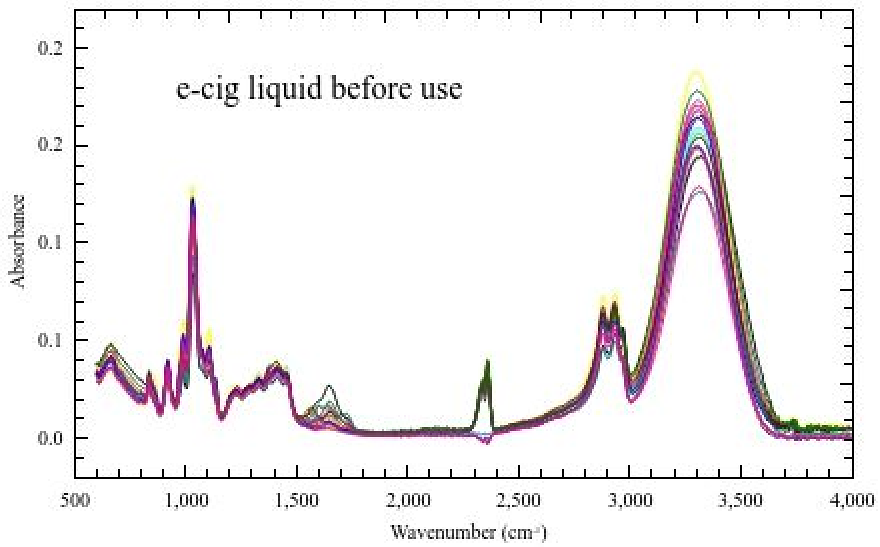

S3. e-cig liquid before use.

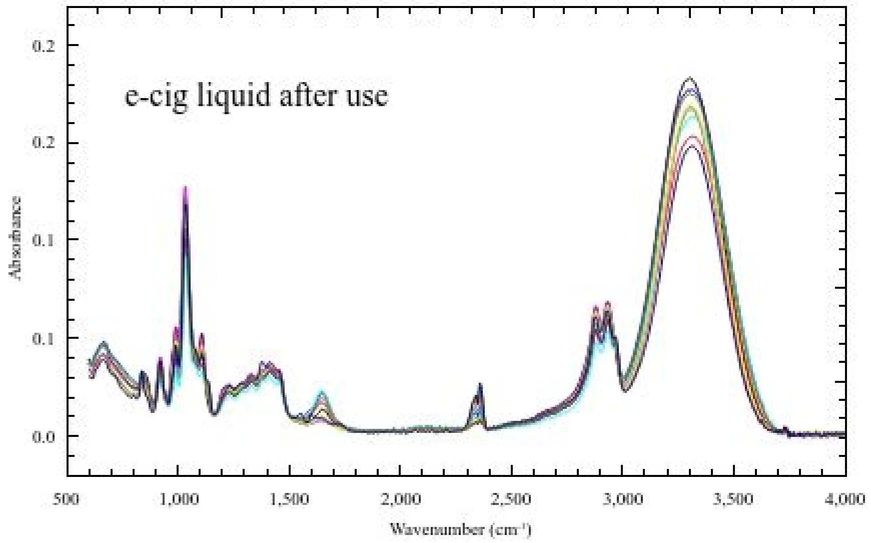

S4. e-cig liquid after use.
